# Exosomes Released from *M.tuberculosis* Infected Cells Can Suppress IFN-γ Mediated Activation of Naïve Macrophages

**DOI:** 10.1371/journal.pone.0018564

**Published:** 2011-04-14

**Authors:** Prachi P. Singh, Christopher LeMaire, John C. Tan, Erliang Zeng, Jeffery S. Schorey

**Affiliations:** 1 Department of Biological Sciences, University of Notre Dame, Notre Dame, Indiana, United States of America; 2 Eck Institute for Global Health, University of Notre Dame, Notre Dame, Indiana, United States of America; 3 Department of Computer Science and Engineering, University of Notre Dame, Notre Dame, Indiana, United States of America; University of California Merced, United States of America

## Abstract

**Background:**

Macrophages infected with *Mycobacterium tuberculosis* (*M.tb*) are known to be refractory to IFN-γ stimulation. Previous studies have shown that *M.tb* express components such as the 19-kDa lipoprotein and peptidoglycan that can bind to macrophage receptors including the Toll-like receptor 2 resulting in the loss in IFN-γresponsiveness. However, it is unclear whether this effect is limited to infected macrophages. We have previously shown that *M.tb*-infected macrophages release exosomes which are 30–100 nm membrane bound vesicles of endosomal origin that function in intercellular communication. These exosomes contain mycobacterial components including the 19-kDa lipoprotein and therefore we hypothesized that macrophages exposed to exosomes may show limited response to IFN-γ stimulation.

**Methodology/Principal Findings:**

Exosomes were isolated from resting as well as *M.tb*-infected RAW264.7 macrophages. Mouse bone marrow-derived macrophages (BMMØ) were treated with exosomes +/− IFN-γ. Cells were harvested and analyzed for suppression of IFN-γ responsive genes by flow cytometry and real time PCR. We found that exosomes derived from *M.tb* H37Rv-infected but not from uninfected macrophages inhibited IFN-γ induced MHC class II and CD64 expression on BMMØ. This inhibition was only partially dependent on the presence of lipoproteins but completely dependent on TLR2 and MyD88. The exosomes isolated from infected cells did not inhibit STAT1 Tyrosine phosphorylation but down-regulated IFN-γ induced expression of the class II major histocompatibity complex transactivator; a key regulator of class II MHC expression. Microarray studies showed that subsets of genes induced by IFN-γ were inhibited by exosomes from H37Rv-infeced cells including genes involved in antigen presentation. Moreover, this set of genes partially overlapped with the IFN-γ-induced genes inhibited by H37Rv infection.

**Conclusions:**

Our study suggests that exosomes, as carriers of *M.tb* pathogen associated molecular patterns (PAMPs), may provide a mechanism by which *M.tb* may exert its suppression of a host immune response beyond the infected cell.

## Introduction

Interferon-γ plays a critical role in host response to *M. tuberculosis* infection [1]. It activates macrophages to control intracellular *M. tuberculosis* (*M.tb*) by inducing the expression of nitric oxide synthase 2 [2] and phagocyte oxidase [3] thus triggering the production of reactive nitrogen and oxygen intermediates respectively. Moreover, it facilitates antigen processing and presentation by macrophages to CD4+ T cells by upregulating MHC class II expression [4]. It has also been reported that IFN-γ induces autophagy which facilitates*M.tb* killing and this is due, at least in part, to the activation of IFN-γ inducible immunity relatedGTPase Irgm1, also known as LRG-47[5], [6], [7]. However, although IFN-γ treatment promotes macrophage's ability to control infection, it has been shown that *M.tb*-infected cells are partially resistant to IFN-γ stimulation. This includes infected macrophages being refractory to IFN-γ killing of *M.tb*. [8]. At least two different mycobacterial components have been identified that inhibit macrophage response to IFN-γ, namely lipoproteins including the 19-kDa lipoprotein and the mycolylarabinogalactanpeptidoglycan complex (mAGP complex) [9], [10], [11]. However, it is unclear if the effect of these mycobacterial components is limited to infected macrophages or whether mycobacterial components can gain access to adjacent uninfected macrophages and if so what is the mechanism involved?

Previous studies by Johnstone and coworkers identified an alternative pathway for extracellular release of transferrin receptors during reticulocyte differentiation [12]. They showed that multivesicular bodies (MVBs) that are formed by the invagination of the limiting endosomal membrane and are known to transport membrane proteins and lipids to the lysosome for degradation, could also fuse with the plasma membrane and release the intraluminal vesicles into the extracellular environment. These vesicles, which have been given the name exosomes, are secreted by cells of hematopoietic and non-hematopoietic origin and are generally thought to function in intercellular communication [13]. It has been shown previously that mycobacterial components including the 19-kDa lipoprotein are trafficked to the MVBs and subsequently released from cells via exosomes [14], [15]. The exosomes released from *M.tb*-infected macrophages can interact with cells of the immune system stimulating macrophage production of pro-inflammatory mediators such as TNF-α as well as activation of naïve antigen-specific T cells *in vivo* [16]. However, exosomes containing mycobacterial components may also modulate macrophage function to promote mycobacterial survival. One possible mechanism in this context is the potential for exosomes to render macrophages refractory to subsequent activation by IFN-γ. Indeed, we found that exposure of naïve macrophages to exosomes derived from *M.tb*-infected cells renders the macrophages refractory to subsequent IFN-γ activation for a subset of genes. This list partially overlaps with IFN-γ-induced genes suppressed by *M.tb* infection and suggest that the ability of *M.tb* infection to suppress IFN-γ stimulation may not be limited to infected cells.

## Results

### Exosomes derived from H37Rv-infected macrophages inhibit IFN-γ-induced MHC Class II and CD64 surface expression on naïve murine macrophages

**Figure 1:**
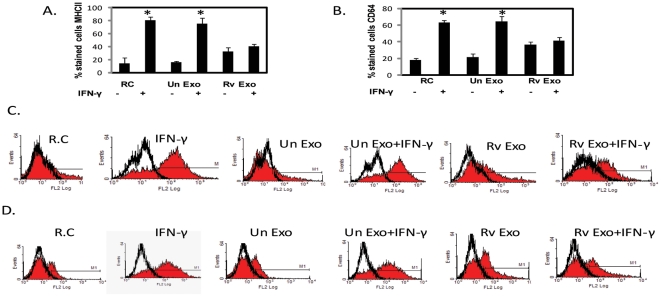
Exosomes isolated from *M.tb* infected cells inhibit IFN-γ induced surface expression of MHC class II and CD64 on BMMØ. Exosomes were isolated from uninfected RAW264.7 cells (Un exo) or RAW264.7 cells infected with *M.tb* H37Rv (Rvexo). BMMØ were treated with exosomes for 18 hours or left untreated (RC) followed by +/− IFN-γ stimulation for an additional 18 hours. Cells were stained with either PE conjugated anti-MHC class II or anti-CD64 and analyzed by flow cytometry. Shown are the number of cells stained with MHC class II (A) or CD64 (B) and representative FACS plots for each treatment; MHC-II (C) and CD64 (D). Isotype control antibody was used to define background staining. Results are representative of three individual experiments plus standard deviations; asterisk (*) indicates a p value ≤0.05 between +/− IFN-γ treatments.

Exosomes were isolated from RAW264.7 macrophages infected with *M. tuberculosis* H37Rv or from uninfected cells. Naïve C57BL/6 bone marrow-derived macrophages (BMMØ) were stimulated with exosomes for 18 hours followed by IFN-γ treatment for an additional 18 hours. Exosomes were removed prior to IFN-γ treatment. The cells were harvested and analyzed for MHC class II and CD64 surface expression by flow cytometry. As expected, treatment of macrophages with IFN-γ markedly upregulated the number of macrophages expressing MHC class II (Fig. 1A and 1B)and CD64 (Fig. 1C and 1D) in comparison to resting cells. Prior treatment with exosomes from infected cells mitigated this IFN-γ-induced MHC class II and CD64 expression (Fig. 1A–D). Exosomes from uninfected cells did not inhibit the MHC class II or CD64 upregulation by IFN-γ nor did treatment with these exosomes alone lead to any significant increase in the surface expression of these proteins. In contrast, exosomes released from infected cells when added to BMMØ increased expression of MHC class II and CD64 approximately 2 fold but no further increase was observed upon IFN-γ stimulation.

### The inhibition of IFN-γ induced MHC class II and CD64 expression by *M.tb*exosomes was partially dependent on the presence of lipoproteins but completely dependent on TLR2 and MyD88

**Figure 2:**
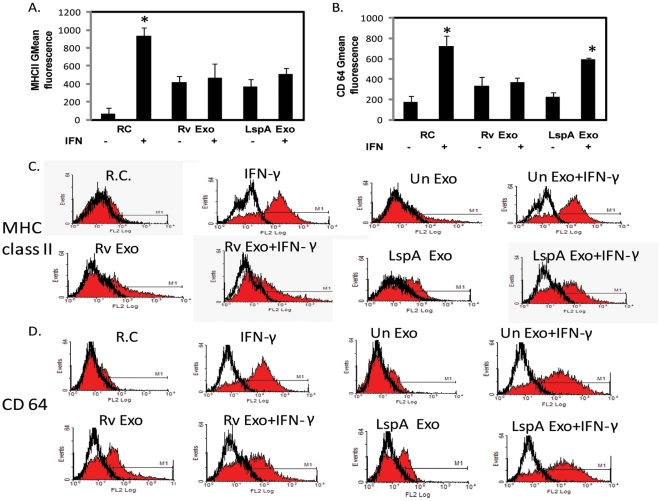
Exosome-mediated inhibition of MHC-II and CD64 expression is partially dependent on exosomes containing mycobacterial lipoproteins. Exosomes were isolated from RAW264.7macrophages infected with wild-type or LspA-deficient *M.tb*. BMMØ were treated with the exosomes or left untreated (RC) for 18 hours and then incubated for an additional 18 hours +/− IFN- γ. Cells were stained with PE-conjugated anti-MHC class II or anti-CD64 antibody and analyzed by flow cytometry. Isotype control antibodies were used to define background staining. Shown is the mean fluorescence intensity for each sample with isotype control values subtracted from each value for MHC-II (A) and CD64 (B). Also shown are the representative FACS plots for MHC-II (C) and CD64 (D) expression. Results are representative of two independent experiments plus standard deviation and p value <0.05 between +/− IFN-γ treatments are indicated by asterisk (*).

It is known that purified *M.tuberculosis* 19-kDa lipoprotein inhibits the induction of a subset of IFN-γ responsive genes through a TLR2 dependent manner. However, live virulent *M.tb* inhibits macrophage response to IFN-γ independent of mature mycobacterial lipoproteins [17]. We therefore hypothesized that exosomes derived from infected cells might also not require lipoprotein. To test this hypothesis, we compared the ability of exosomes released from cells infected with either wild-type or LspA-deficient H37Rv to inhibit the IFN-γ-induced surface expression of MHC-II and CD64. The *lspA*gene encodes for a prolipoprotein signal peptidase II that cleaves the signal sequence from diacylatedprolipoproteins at a site that precedes the lipidatedcysteine residue. This cleavage exposes the primary amine group on the N-terminal cysteine that leads to final acylation and formation of mature triacylated lipoproteins. Disruption of lipoprotein signal peptidase results in loss of all mature lipoproteins. We found that exosomes from H37Rv *lspA^-^* infected cells alone induced a limited increase in MHC class II expression similar to what was observed for exosomes from wild-type H37Rv-infected cells. Moreover, both exosome preparations inhibited the IFN-γ induced MHC class II expression (Fig. 2A and 2C). In contrast, the exosomes from H37Rv *lspA^-^* infected cells neither induced CD64 expression nor were they able to inhibit the IFN-γ-induced CD64 expression, suggesting differential inhibition of IFN-γ responsive genes in presence/absence of mycobacterial lipoproteins (Fig. 2B and 2D). The reason for this difference is not clear but may reflect a quantitative difference in TLR2 ligation in the presence or absence of lipoproteins. This may lead to differences in the level of NF-κB activation or other signaling changes resulting in the distinct responses observed with the exosomes.

**Figure 3:**
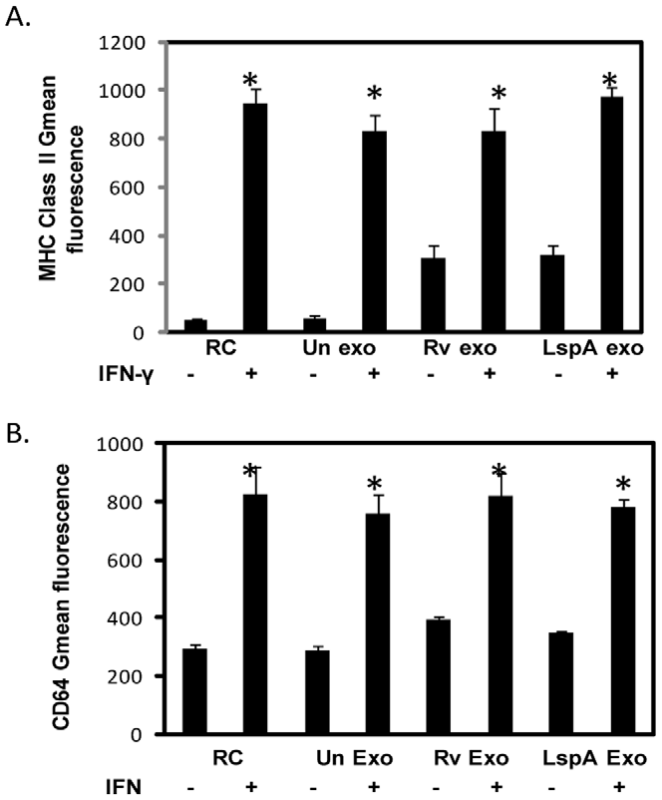
Theinhibition of MHC class II and CD64 by Rvexosomesis dependent on macrophage expression of TLR2. Untreated or exosome-treated BMMØ isolated from TLR2-deficient mice were stimulated +/− IFN-γ. Macrophages were harvested, stained with PE-conjugated anti-MHC class II or anti-CD64 antibody and analyzed by flow cytometry. Shown are the mean fluorescence intensity values for MHC-II (A) and CD64 (B) expression. Results are representative of two independent experiments plus standard deviation and p value <0.05 between +/− IFN-γ treatments are indicated by asterisk (*).

Nevertheless, exosome-mediated suppression of IFN-γ-induced MHC class II and CD64 expression was completely dependent on TLR2 (Fig. 3A and 3B) and MyD88 (data not shown) as macrophages deficient in either protein were refractory to the exosome-mediated inhibition of IFN-γ activation. Interestingly, the limited upregulation of MHC class II expression induced by exosomes was not dependent on TLR2 (Fig. 3A).

Since *M.tb-*infected macrophages secrete IL-6 which may contribute to their inhibition of IFN-γ activation [18], we tested the cell culture supernatants from exosome-treated BMMØ for IL-6 levels. We found that although treatment of BMMØ with exosomes from infected cells resulted in higher levels of IL-6 in comparison to treatment with exosomes from uninfected cells, neither supernatants were capable of suppressing IFN-γ-induced MHC-II and CD64 expression (data not shown).

### Exosomes from *M.tb*-infected cells do not block IFN-γ induced STAT1 phosphorylation but do block CIITA expression

**Figure 4:**
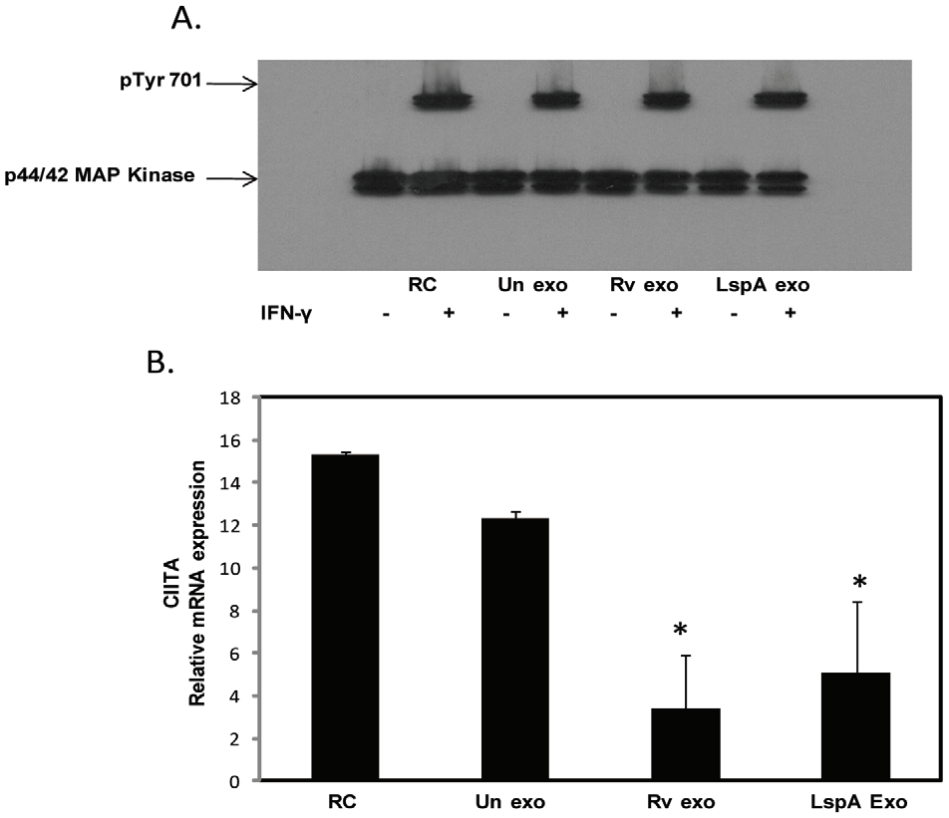
Exosomes from *M.tb*-infected cells do not block IFN-γ induced STAT1 phosphorylation but do inhibit IFN-γ induced expression of CIITA. BMMØ were treated +/− exosomes isolated from RAW264.7 macrophagesas described for figure 1 followed by a 30 minute incubation with IFN-γ. Cells were lysed and analyzed by Western blot for p-STAT1 (Tyr701) (A). The p44/42 MAP Kinase antibody was used as a loading control as described previously (17). BMMØ were treated with exosomes and stimulated +/− IFN-γ for 18 hours. Cells were harvested for qRT-PCR using specific primers for target gene (CIITA) and reference gene (GAPDH). Shown is the relative mRNA expression compared to untreated cells for CIITA normalized to GAPDH (B). Results are representative of two separate experiments plus standard deviation and p value <0.05 shown by asterisk (*).

To determine if exosomes block the initial steps in the JAK-STAT pathway we looked at STAT1 phosphorylation following IFN-γ stimulation. As observed previously with *M.tb* and its TLR2 ligands [19], [20], we found that exosomes from wild-type or *LspA^-^* H37Rv-infected cells did not inhibit phosphorylation of STAT1 at Tyr 701 (Fig. 4A). Previous studies have shown that the *M.tb* 19-kDa lipoprotein inhibits class II major histocompatibity complex transactivator (CIITA) expression normally induced by IFN-γ treatment [20]. CIITA is a master regulator of MHC class II expression and appears to promote transcription factor binding to the MHC class II promoter facilitating transcription [21]. To determine whether pre-treatment of macrophages with exosomes also blocks IFN-γ-induced CIITA expression, real time PCR was performed to measure CIITA mRNA levels. The results showed that exosomes from wild-type or *lspA^-^* H37Rv-infected cells inhibited IFN-γ induced CIITA mRNA expression by 5 fold and 3 fold respectively in comparison to treatment with IFN-γ alone (Fig. 4B). The CIITA mRNA levels on macrophages were defined relative to the housekeeping gene GAPDH. Exosomes from uninfected cells did not inhibit the IFN-γ induced CIITA mRNA expression (Fig. 4B).

### Exosomes from *M.tb*-infected cells inhibit a subset of IFN-γ regulated genes

**Figure 5:**
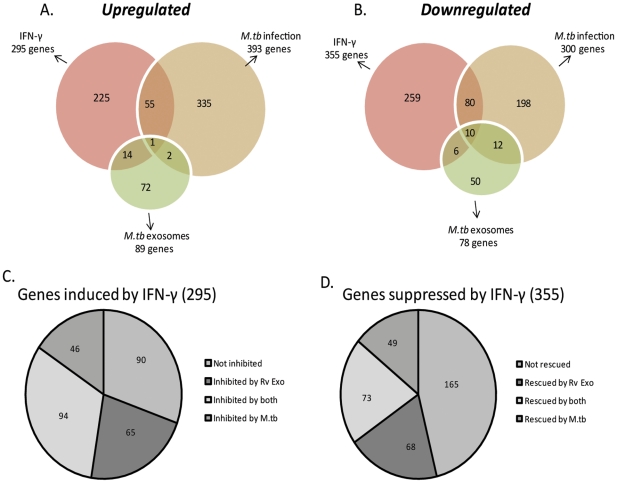
Exosomes isolated from *M.tb*-infected cells inhibit a subset of IFN-γ inducible genes which partially overlaps with those inhibited by an *M.tb* infection. BMMØ were treated with exosomes isolated from RAW264.7 cells (uninfected/infected*)*or were infected with *M.tb* followed by incubation +/− IFN-γ. RNA was isolated, converted to double-stranded cDNA, labeled with Cy3 and hybridized to *Musmusculus* 4×72 whole genome array. Genes up-regulated or down-regulated by each treatment were identified on the basis of ≥2 fold change in gene expression and a p value ≤0.05 as defined through three independent microarray experiments. Results are represented as Venn diagrams showing the total number of genes identified as well as the number of genes which overlap between treatment groups (A and B). Genes induced by IFN-γ were further analyzed for suppression by exosome pre-treatment or H37Rv infection. Similar analysis was performed on genes suppressed by IFN-γ whose expression was “rescued” by exosome pre-treatment or H37Rv infection. Results are depicted as pie charts showing the number of IFN-γ-induced genes not inhibited by any treatment, those inhibited by treatment or infection and those common to both groups (C). Similarly, results are shown for genes rescued by exosomes or H37Rv infection (D).

A microarray study was undertaken to define the gene expression profile in response to exosome treatment on a global scale. Primary C57BL/6 derived BMMØ were treated with exosomes or infected with *M.tb* followed by incubation with or without IFN-γ. There were 8 treatment groups which included BMMØ: +/− IFN- γ, Uninfected exosomes +/− IFN-γ, H37Rv exosomes +/− IFN- γ and H37Rv infection +/− IFN- γ. Macrophages were harvested for RNA which was converted to double stranded cDNA, labeled with Cy3 and 4 µg of labeled cDNA hybridized to *Musmusculus* 4×72 whole genome array. Results were drawn from three independent experiments and expression values for treatment groups were normalized to untreated macrophages. Genes were filtered on the basis of fold change ≥2 and p value ≤0.05. Treatment of macrophages with IFN-γ induced the expression of 295 genes and suppression of 355 genes (Fig. 5A and B). Included in the upregulated genes are those involved in antigen presentation such as MHC-II and CD64 as well as CD40, CIITA, CD86 and H2-Mb2. Other genes upregulated by IFN-γ include those involved in cell attachment and recruitment (e.g. ICAM-1, CCR2 and CCR5) and GTP binding proteins (e.g. guanylate binding proteins 2, 4 and 5, IRG-47, LRG-47 and Interferon-γ induced GTPase). Infection of macrophages with *M.tb* resulted in the induction of 393 genes and suppression of 300 genes whereas treatment with exosomes from H37Rv-infected cells induced and suppressed 89 and 78 genes respectively. A number of genes suppressed by exosomes are involved in immune responses such as the nitric oxide synthase, PGE2 synthase and HSP-70. Exosomes from uninfected cells induced the expression of 5 genes that were all listed as hypothetical and no suppressed genes were defined. *M.tb* infection induced expression of many the same genes previous defined [22] including Formyl peptide receptor, SOCS-3, TRAF-1 and Fas as well as suppressed expression of Ccr2, Cyclin D1, Lymphocyte specific protein, CD39 and CD83. There was a limited overlap in up-regulated and down-regulated genes between any two treatments with most of overlap observed between IFN-γ treatment and H37Rv infection. A complete list of the microarray data associated with IFN-γ, *M.tb*-infection and exosome-treatment of BMMØ has been included as supplementary data (TableS1) and submitted to Gene Expression Ominbus (GEO).

In this study, we were particularly interested in evaluating how exosomes affect the macrophage response to IFN-γ and how this compares to *M.tb*-infected macrophages. We observed that of the 295 genes induced by IFN-γ, 90 were not inhibited by any treatment or infection (Fig. 5C). However, there were a significant number of IFN-γ inducible genes (94 in total) that were suppressed by either pretreatment with H37Rv exosomes orH37Rv infection. Interestingly, an additional 65 genes induced by IFN-γ, were specifically suppressed by H37Rv exosomes and similarly 46 IFN-γ inducible genes were uniquely suppressed in H37Rv infected cells. Importantly, pretreatment of macrophages with exosomes released from uninfected cells did not significantly affect any of the genes induced by IFN-γ. We also analyzed the genes suppressed by IFN-γ to determine if pre-treatment with H37Rv exosomes or prior infection with *M.tb*“rescued” gene expression. We found that among the 355 genes suppressed by IFN-γ, a significant number of genes (165 in total) were not rescued by exosome treatment or infection (Fig. 5D). However, there were a number of genes (73 in total) rescued by either pretreatment with H37Rv exosomes or by *M.tb* infection. An additional 68 genes were rescued only in BMMØ treated with H37Rv exosomes and another 49 genes were rescued only in *M.tb*-infected cells. Again, pretreatment with exosomes from uninfected cells had no effect on IFN-γ regulated gene expression. A complete list of the microarray data associated with IFN-γ-regulated genes affected by exosomes treatmentand *M.tb* infection can be found in supplementary data (Table S2) and has been submitted to GEO.

Table 1 shows a list of IFN-γ- regulated genes whose expression are affected by H37Rv exosome treatment and includes the genes most altered by exosome treatment based on fold differences in gene expression. Also included is a subset of genes likely associated with *M.tb* immunity taken from the complete list of IFN-γ- regulated genes modulated by H37Rv exosome treatment. These genes fall into various categories including genes important in antigen presentation (i.e CIITA, class II MHC, CD86), apoptosis (i.eFas, caspase 3) and complement components. Of note, the majority of the genes listed were also suppressed by *M.tb*infection. We were particularly interested in the exosome-mediated suppression of *Irgm* mRNA expression which was confirmed by qRT-PCR (data not shown). Irgm is a GTP-binding protein which appears to play a critical role in the anti-mycobacterial response induced by IFN-γ [5].

**Table 1:** List of genes suppressed or rescued by exosomes isolated from *M.tb* infected cells following stimulation with IFN-γ.

*IFN- γ inducible genes suppressed by Rvexosomes*	*Genes rescued by Rvexosomes from IFN- γ mediated suppression*		
Ten most regulated genes			
Gene	Fold change	Gene	Fold change
Cnn3, calponin 3, acidic	−21.67*	Edg1,endothelial differentiation	15.06*
Bcl2l14, Bcl-2 like14	−17.09*	sphingolipid G-protein coupled receptor1	
(apoptosis facilitator)		Ccl9	11.75*
Mpa2l,macrophage activation 2 like	−14.49*	Clec4d, C-type lectin domain4	11.03*
Cxcl10,(chemokine C-X-C motif)	−13.15*	Lhfpl2,lipoma HMGIC	10.7*
Ligand 10		fusion partner like 2	
Cd274	−9.54*	Igf1, insulin like growth factor1	10.28*
Slamf8,SLAM family member8	−7.39*	Fabp4,fatty acid binding protein4	10.14*
Gbp5,guanylate nucleotide binding protein5	−7.04*	Igfbp4,insulin like growth factor binding protein4	9.75*
Lrrc8c, Leucine rich repeat containing 8 family member C	−6.96*	cbr2, carbonyl reductase2	7.78*
TimD4,T-cell immunoglobulin	−6.82*	Emp1,epithelial membrane protein1	7.28*
Cd40	−6.70*	Xylt2, xylosyltransferase2	6.68*

Ten most interesting genes			
C2ta, classIItransactivator	−4.41*	C5ar1,complement component 5a receptor1	4.3*
Cd86	−3.55*	Cd14	4.19*
Fas, Tnfrsf6 (TNF receptor superfamily member)	−3.38	Mrc1, mannose receptor C,type1	4.04
H2-DMb2, histcompatibility2,classII, locusMb2	−3.08*	Ccr3	3.98*
irf1,interferon regulatory factor1	−2.87*	Pik3cg	3.34*
Ccl5, RANTES	−2.97	Cd84	3.24*
irgm, LRG-47,immunity related GTPase	−2.35*	Ccl3	2.90*
Caspase3	−2.43	Ccl4	3.1*
C3, complement component 3	−2.62	Ets-1	2.25*
C1r, Complement component1,r	−2.25	Cxcr4	4.44*

*Genes common to both treatment with Rv exosomes/H37Rv infection.

To categorize the differentially regulated genes functionally, we initiated a KEGG pathway analysis using web based Pathway-Express program of the Onto-tools Suite. This led to identification of 6 major pathways that were down-regulated in cells treated with H37Rv exosomes and IFN-γ compared to IFN-γ treatment alone (Table 2). These pathways were selected on the basis of corrected gamma p value ≤0.05 and a minimum input of 4 genes. Most significantly affected pathways included those involved in Cell adhesion and Cytokine-cytokine receptor interaction. Other affected pathways include the antigen processing and presentation pathway, Toll-like receptor signaling pathway, apoptosis and complement and coagulation pathway.

**Table 2:** IFN-γ stimulated pathways significantly inhibited by Rvexosomes.

Pathway	No of genes	Gamma p value	Representative genes
Cell adhesion molecules	10	2.26E-06	Itgb7, CD40, CD274, CD86, Icam1
Cytokine-cytokine receptor interaction	10	9.11E-05	Il15ra, il18, ccl12, ccl5, cxcl10
Antigen processing and presentation	6	0.001	H2-T22, ciita, H2- DMb2, H2-Oa,Tap1, H2-DMa
Toll-like-receptor signaling pathway	8	0.002	Irf-7, tnf, CD40, Tlr9
Complement and coagulation cascades	4	0.003	Clr, c3, c1s, cfb
Apoptosis	7	0.005	Fas, TNF, Casp3, Bid

## Discussion

Our results suggest that exosomes released from *M.tb* H37Rv-infected cells when added to BMMØ can partially block the cells response to IFN-γ stimulation, including inhibiting MHC class II expression. The mechanism by which exosome inhibit the IFN-γ response appears similar to what has been shown with the 19-kDa lipoprotein and *M.tb* infection, as the proximal step in STAT1 activation appear not to be affected but expression of CIITA is down-regulated [19], [20], [22], [23], [24], [25]. This inhibitory effect of exosomes is partially dependent on the presence of *M.tb* lipoproteins but completely dependent on the macrophage expression of TLR2 and MyD88. Finally, in line with previous studies with *M.tb*-infected macrophages or purified lipoprotein, the exosomes only block a subset of IFN-γ inducible genes [22].

Although a significant body of research has addressed the mycobacterial components that can inhibit IFN-γ stimulation as well as define the mechanism behind this inhibition [9], [10], [11], [20], [22], [25], it remains unclear whether these components would be acting only in the context of whole mycobacteria and therefore only within infected cells or if they may function outside this sphere. The latter requires that the components are shed while the mycobacteria remain extracellular or that they are released from infected cells. Although, published data indicates that *M.tb* may remain extracellular during a latent infection [26], studies suggest that only limited number of bacilli remain extracellular during an active infection [27]. Nevertheless, it is clear that mycobacterial components can be found extracellular. Cord factor can be found at high concentrations within TB granulomas in human patients and likely functions in promoting caseatinggranulomas and secondary tuberculosis [28]. How cord factor accumulates in the extracellular environment is unclear, although experiments by Beatty *et al.* indicate that it is released from infected macrophages on small membrane-bound vesicles [29]. However, most studies to address mechanisms by which mycobacterial components are released from infected cells have focused on apoptosis. Apoptotic vesicles isolated from BCG-infected macrophages can activate both CD4+ and CD8+ T cells *in vivo* [30]. Earlier studies have shown that apoptotic bodies from infected macrophages carry a number of mycobacterial antigens [31]. Our recent work indicates that mycobacterial proteins and glycolipids are present on exosomes released from macrophages infected with *M.tb*or other mycobacteria [32].

Exosomes are small 30–100 nm membrane vesicles derived from the fusion of MVBs with the plasma membrane and release of the intraluminal vesicles as exosomes. The exosomes are released from both hematopoietic and non-hematopoietic cells and function in transporting proteins, lipids and RNA to other cells and in doing so may modulate their cellular functions [33]. The role for exosomes in biological processes has garnered considerable attention recently, mostly in the context of tumor antigen presentation and activation of CD4+ and CD8+ T cells [34], [35], [36]. However, exosomes have also been shown to inhibit immune responses. Studies by Peche*et al*. demonstrated that by injecting donor-haplotypeexosomes from bone marrow DCs before transplantation one could significantly prolong heart allograft survival in congenic and fully MHC mismatched Lewis rats [37]. Exosomes may also be involved in promoting tolerance to oral antigens [38]. Therefore, whether exosomes promote or suppress an immune response depend on various factors including source and composition of the exosomes as well as the system under investigation. In previous studies we have shown that exosomes from *M.tb*-infected macrophages can stimulate naïve macrophages to produce limited amounts of TNF-α, IL-6 and other pro-inflammatory mediators [15]. In the present study we have shown they can also enhance MHC class II cell-surface expression. Nevertheless, pre-treatment of BMMØ with exosomes from *M.tb*-infected but not from uninfected cells can render these macrophages partially refractory to IFN-γ stimulation.

In previous studies by Pai*et al*. they observed a significant number of macrophage/host genes whose expression was modulated by exposure to the *M.tb* 19-kDa lipoprotein (459 genes in total). Interestingly, despite the known presence of the 19-kDa lipoprotein on exosomes, we did not observe such modulation of macrophage gene expression upon exposure to exosomes from *M.tb*-infected macrophages (167 genes in total). This might be due to differences in the concentration of the 19-kDa lipoprotein as the exosomes would likely contain significant less of the lipoprotein then used in the studies by Pai*et al*. The variation may also stem from differences in the experimental systems, as the previous microarray studies used macrophages exposed to the 19-kDa lipoprotein during the entire experiment while in our studies, the BMMØ were exposed to exosomes only during the first 18 hours and removed during the 18 hour incubation with IFN-γ. It is important to note that our study was not specifically designed to look at early changes in gene expression induced by exosomes. Nevertheless, it is clear that exosomes can modulate macrophage gene expression and that this is specific to exosomes from infected cells as exosomes from uninfected macrophages were relatively inert. As expected, infection of macrophages with H37Rv induced significant changes in gene expression and was comparable to what was observed in earlier studies [22].

Our study indicates that exosomes from *M.tb*-infected macrophages can block a significant number of IFN-γ-induced or suppressed genes. A majority of these IFN-γ regulated genes were also blocked in *M.tb*-infected macrophages and include genes involved in antigen presentation and macrophage activation (see Table 1). Indeed all 10 genes which showed the highest change in gene expression between IFN-γ treatment +/− exosomes were also affected by infection with *M.tb*. Nevertheless, there were also IFN-γ regulated genes specifically affected by treatment with exosomes or infection with H37Rv. This likely stems from the different receptors engaged by H37Rv and exosomes and the continued presence of the mycobacteria and mycobacterial components during the course of the experiment. Our previous studies indicate that exosomes function through TLR2 and MyD88 to induce macrophage production of TNF-α and other cytokines [15] and this engagement also appears important in modulating the macrophage response to IFN-γ; however, we cannot rule out the importance of other receptors potential engaged by exosomes.

In summary, the present as well as our previous studies suggest that the exosome's effect on the immune response is dynamic and multifactorial. Exosomes released from *M.tb*-infected macrophages can both promote and inhibit aspects of mycobacterial immunity. To what extent exosomes perform such functions during the course of an *in vivo* infection awaits further study and will require manipulation of both the production and composition of exosomes during an infection.

## Materials and Methods

### Ethics Statement

The University of Notre Dame is credited through the Animal Welfare Assurance (#A3093-01). All animal studies were conducted according to the Institutional Animal Care and Use Committee (IACUC) guidelines. The protocol for the isolation of macrophages from mice was approved by the University's IACUC (March 26, 2009, protocol # 11-034).

### Macrophage culture and bacterial strains

Bone marrow derived macrophages (BMMØ) were isolated from 6–8 weeks old female C57BL6 mice and cultured *in vitro* as previously described [39]. The mouse macrophage cell line RAW 264.7 was maintained in RPMI supplemented with 10% fetal bovine serum, 10 mM sodium pyruvate and 25 mM HEPES. wild-type and the LspA-deficient H37Rv (kindly provided by Joel Ernst, NYU, New York) were each grown in Middlebrook 7H9 broth supplemented with OADC until mid logarithmic growth phase and frozen down as stocks in growth media plus 15% glycerol. Prior to use, H37Rv stocks were thawed and the mycobacteria were de-clumped bya brief sonication and passing through syringe fitted with 27 gauge needle at least ten times.

### Isolation of exosomes from cell culture supernatants

Confluent monolayers of RAW 264.7 mouse macrophage cell line were infected with mycobacteria or left uninfected as controls. Before infection, the bacterial cultures were incubated for 2 hours with normal horse serum for complement opsonization. Infections with *M.tb* were titrated to obtain approximately 80% infectivity. The RAW 264.7 cells were infected with bacteria for 4 hours followed by washes with 1X DPBS. The cells were cultured in RPMI containing exosome free FBS (10% final concentration) and exosomes were isolated from the culture supernatants of infected and uninfected RAW 264.7cells after 72 hours and purified on linear sucrose gradient as previously described [40].

### Treatment of macrophages with exosomes and IFN-γ

BMMØ were seeded in six well tissue culture plates @ 7.5×10^5^ cells/well and allowed to adhere for 24 hours. The cells were treated with exosomes isolated from uninfected and infected RAW cells at 10 µg/well for 18 hours. The media was removed and replenished with fresh media +/− recombinant mouse IFN-γ (eBioscience Inc. San Diego, CA) at 200 U for an additional 18 hours.

### Flow cytometry

The cells were rinsed with DPBS and gently scraped and counted on hemacytometer using trypan blue to assess viability. The cells were washed in FACS buffer and blocked with 10% mouse serum and stained with PE conjugated anti-mouse I-A/I-E or FcγR1 (CD 64) or isotype control (BD Pharmingen, San Jose, CA). Cells were analyzed for protein surface expression using a Beckman Coulter flow cytometer. Cellular fluorescence was also quantified by the geometric mean (Gmean). For this fluorescence analysis, the GMean fluorescence for isotype control was deducted from the GMean fluorescence for the specific antibody.

### Immunoblot

To study phosphorylation of STAT1, the bone marrow macrophages were treated with exosomes for 18 hours as described above. Cells were treated with 200 U recombinant mouse IFN-γor left untreated for 15 minutes to an hour. Cells were then lysed on ice in immunoprecipitation buffer with protease and phosphatase inhibitors (20 mMTris.HCl, 50 mMNaCl, 1 mM EGTA, 1% NP-40, 1% Sodium deoxycholate, 2.5 mM sodium pyrophosphate, 1 mM sodium vanadate, 1 mM EDTA, 1 mM Dithiothreitol, 1 mM PMSF 0.1% SDS and 10 mM sodium fluoride). The protein lysates were quantified by Micro BCA protein assay and equal concentrations of each group were loaded on 10% SDS-PAGE gels, electrophoresed and transferred onto polyvinylidenedifluoride membranes (Milipore, Bedford, MA). The membranes were probed with (1/1000 dilution) p-Stat1 (Tyr 701) (Santa Cruz Biotechnology Inc. Santa Cruz, CA) and with goat anti-mouse HRP (1/15000 dilution) and developed using Super Signal West Pico chemiluminiscent substrate (Pierce, Thermo Fisher Scientific, Rockford, IL). The p44/42 MAP Kinase antibody (Cell Signaling Technology Inc. Beverly, MA) was used as a loading control as previously described [41].

### Quantitative Real time PCR

RNA was isolated using RNeasy columns (Qiagen Inc. Valencia, CA) following manufacturer's instructions and quantified by Nanodropspectrophotometric method. 1 µg of RNA was converted to cDNA using Verso cDNA kit (Thermoscientific). One tenth of the resulting cDNA template was used for Real-time PCR using Absolute QPCR SYBR Green Mix (Thermo Scientific) and 7500 Fast Real Time PCR system (Applied Biosystems). The following primers were obtained commercially from Invitrogen: CIITA sense, 5′-ACG CTT TCT GGC TGG ATT AGT-3′; CIITA antisense,5′-TCA ACG CCA GTC TGA CGA AGG-3′ and GAPDH sense, 5′-AAC GAC CCC TTC ATT GAC-3′, GAPDH antisense,5′- TCC ACG ACA TAC TCA GCAC-3′ [25]. PCR amplification efficiencies were determined for each gene prior to the relative quantification and were similar for the target gene (CIITA) and the endogenous control (GAPDH). Dissociation curve analysis was also run for each reaction to detect nonspecific amplification in cDNA samples. The relative mRNA expression of the target gene normalized to the endogenous reference gene was quantitated using the comparative C_t_ method and the formula 2^-ΔΔC^_T_.

### Nimblegen Microarray gene expression studies

Macrophages were treated with exosomes at10 µg/well, infected with *M.tb* H37Rv or left untreated for 18 hours followed by +/− IFN-γ at 200 U/ml for an additional 18 hours. Cells were harvested and RNA was isolated using RNeasy columns (Qiagen). Double stranded cDNA was synthesized using the Invitrogen Superscript Double-Stranded cDNA synthesis kit and was subsequently labeled using Nimblegen one color DNA labeling kit (Roche Nimblegen Inc. WI). 4 µg of Cy3-labeled cDNA from each group was hybridized onto *Musmusculus* 4×72 Nimblegen microarray using Nimblegen Hybridization system 4 according to manufacturer's instructions (Roche). Arrays were scanned and chip images were collected on a Nimblegen MS200 station running Nimblegen 1.0 software. The microarray gene expression analysis was performed using data generated from three independent experiments. All genes that fell within one standard deviation of the average chip background in at least two thirds of treatment conditions were removed from consideration before any other analysis was performed. P values for differences in individual gene expression values were calculated using the student t-test. Separate gene lists were constructed for up- and down-regulated genes for each of the three treatment conditions by screening with the criteria of mean expression value of ≥2 fold over untreated cells and a p value ≤0.05. From the list of IFN-γ induced genes (determined by the above criteria) a gene was considered suppressed by exosome treatment or H37Rv infection if it showed at least 2 fold down regulation relative to IFN-γ treatment with p value <0.05. Similarly, an IFN-γ suppressed gene was considered rescued if it showed at least 2 fold increase in expression level relative to IFN- γ treatment with a p value <0.05.

Pathway analysis was performed with the Pathway-Express program [42] of the Onto-tools Suite. For this analysis, genes were selected based solely on the criterion of a 2 fold cut-off. The analysis was performed assuming a hypergeometric distribution and using the Bonferroni multiple testing correction. Affected KEGG pathways were ranked in order of classical p-values as well as gamma corrected p-values, which refers to pathway impact analysis or a measure of the probability of a pathway being significantly regulated.

#### Statistical analyses

Data was analyzed by a one-tailed or paired Student's t test. Statistical significance was assumed at p ≤0.05. Each experiment was conducted 2 or 3 times and error bars represent standard deviation values.

## Supporting Information

Table S1 List of genes suppressed or rescued by exosomes isolated from *M.tb* infected cells following stimulation with IFN-γ.
(XLSX)

Table S2 IFN-γ stimulated pathways significantly inhibited by Rvexosomes.
(XLSX)
